# Optimization of washing and cooking processes of rice for Ochratoxin A decrement by RSM

**DOI:** 10.1002/fsn3.860

**Published:** 2018-10-29

**Authors:** Rezvan Mansouri‐Nasrabadi, Jafar Mohammadzadeh Milani, Saman Seyed Jafar Nazari

**Affiliations:** ^1^ Department of Food Science and Technology Sari Agricultural Sciences and Natural Resources University Sari Iran; ^2^ Department of Chemistry University of Mazandaran Babolsar Iran

**Keywords:** cooking, Ochratoxin A, rice, washing

## Abstract

In this research, effects of washing and cooking processes on the decrease in ochratoxin A (OTA) residue in rice were investigated. Rice samples were washed one, two, and three times in the washing stage. Results showed that the washing stage was effective on OTA residue as 42.68% of OTA was removed. In the cooking stage, the effects of boiling time, salt content, and water‐to‐rice ratio on OTA residue were studied employing response surface methodology (RSM). The results showed that time and salt content, interaction between time and salt content, and interaction between salt content and water‐to‐rice ratio were effective on the amount of OTA residue. According to the results, the optimum levels for boiling time, salt content, and water‐to‐rice ratio were 9.6 min, 3.5% salt, and 4:1, respectively. At these conditions, 76% of the OTA in rice was reduced.

## 1 INTRODUCTION

Rice is considered as a source of minerals, nutrients, vitamins, and complex carbohydrates (Koesukwiwat, Sanguankaew, & Leepipatpiboon, 2014) and one of the most frequently consumed cereals in the world. Not surprisingly, it provides 20% of the world's dietary energy supply (Lai, Liu, Ruan, Zhang, & Liu, 2015). Therefore, the United Nations has launched a major international drive to increase production of rice, the staple food for more than half of the world's population, and introduced 2004 as the international year of rice (US Department of State, 2003).

Rice kernels can be contaminated by molds during cultivation and subsequent handling. In addition, molds can grow and produce mycotoxins if conditions are favorable. As two important factors affecting fungal activity, moisture content and temperature significantly are dependent on environmental factors in a silo. Adequate drying and storage conditions, as a postharvest treatment of rice, are critical factors determining storage stability (Feizy, Beheshti, Fakoor Janati, & Khoshbakht Fahim, 2011). FAO reported in 2006 that rice harvest waste per year is about 15%–16%, more specifically during crucial postharvest processes (Dors, de Almeida Pinto, & Badiale‐Furlong, 2009).

In 1965, OTA was first discovered from an *Aspergillus ochraceus* isolate (Varga, Kocsubé, Péteri, Vágvölgyi, & Tóth, 2010). OTA is a chlorinated isocoumarin mycotoxin, mainly produced by toxigenic and filamentous fungi (*P. verrucosum*,* A. ochraceus*,* A*. *niger,* and *A*. *carbonarius*) in food and feed (Feizy et al., 2011; Heshmati, Zohrevand, Khaneghah, Nejad, & Sant'Ana, 2017; Iha, Trucksess, & Tournas, 2009; Varga et al., 2010). OTA exists in agricultural products such as cereals, grapes, coffee, spices, cocoa, and their derivatives because of its thermostability (Ali, Hashim, & Shuib, 2015; Ghali, Hmaissia‐Khlifa, Ghorbel, Maaroufi, & Hedili, 2008; Varga et al., 2010). According to the experimental studies on OTA carcinogenicity in animals, the International Agency for Research on Cancer classified OTA as a possible human carcinogen (group 2B, IARC 1993). The toxic effects of OTA appear to be related to its ability to inhibit protein synthesis by competing with phenylalanine in the reaction catalyzed by phenylalanyl‐tRNA synthetase and other systems that require this amino acid. On the other hand, OTA increases lipid peroxidation, resulting in mitochondrial and cell damage increase (Turner, Subrahmanyam, & Piletsky, 2009). Also, this mycotoxin is known to be nephrotoxic, cytotoxic, carcinogenic, teratogenic, and immunosuppressive (Ali et al., 2015).

The European Commission has determined limits of OTA in cereals and derivate products as follows: 5 ng/g for raw cereal grains, 3 ng/g for derivate products intended for human consumption, and 0.5 ng/g for baby food and cereal‐based food intended for young children (EC., 2006). The natural occurrence of OTA has been reported from temperate subtropical and tropical climates in several foods including rice (Juan, Zinedine, Idrissi, & Mañes, 2008). The variety of food processes that may have effects on mycotoxins include cleaning, milling, brewing, cooking, baking, frying, roasting, flaking, nixtamalization, and extrusion. Most of the food processes have variable impacts on mycotoxins, with those that utilize high temperatures having the greatest effects (Karlovsky et al., 2016; Milani & Maleki, 2014). It was reported by Iha et al. (2009) that all processing methods (washing, soaking, and cooking) reduced the OTA amount in beans. The combination of washing, soaking, and cooking facilitated the greatest reduction of OTA residue in beans if the liquid was decanted after each treatment (Iha et al., 2009). OTA concentration, as determined by high performance liquid chromatography (HPLC) analysis, in the rice cooked by cookers was significantly lower than (59%–75%) those of raw polished rice, water‐washed rice, and washing polished rice, with water had little effect on OTA levels (Park, Chung, Lee, & Kim, 2005). Autoclaving oatmeal with 50% water gave a 74% reduction in ochratoxin, while autoclaving dry oatmeal or rice cereal gave more losses as much as 86%–87.5% (Trenk, Butz, & Chu, 1971).

There are some published reports on the OTA reduction in rice products, which concentrated on washing and heating time. The salt is usually added to rice cooking water, which could impact on OTA decontamination. Correspondingly, optimization of boiling time, salt content, and water‐to‐rice ratio on the OTA residue has not considered in previous studies. This study was designed to optimize the cooking processes of the rice to the most decrement of OTA content in rice.

## 2 MATERIALS AND METHODS

### 2.1 Materials

The rice sample (Sadri Hashemi variety) was purchased from retail markets in Mazandaran. OTA standard was obtained from Sigma (St. Louis, MO). Acetonitrile and HPLC‐grade methanol were purchased from Merck (Darmstadt, Germany). OchraTest clean‐up step immunoaffinity chromatography columns (IACs) were supplied by Vicam (Neogen Europe, Scotland, UK). Phosphate‐buffered saline (PBS) was obtained from Panreac (Panreac Química SA, Spain).

### 2.2 HPLC conditions

High performance liquid chromatography analyses were performed by Knauer Smartline 1100 (Knauer, Germany). HPLC system was equipped with an S4011 column thermo‐controller, a RF‐10Axl fluorescence detector, and a Chromolith Performance RP18 analytical column (250 × 4.6 mm, 5 μm). The fluorescence detector was operated at 330 and 470 nm for excitation and emission, respectively. The mobile phase was a mixture of 45% v/v acetic acid–water (98:2) and 55% acetonitrile (Majeed, Iqbal, Asi, & Iqbal, 2013).

### 2.3 Spraying of rice sample by OTA

According to Commission Regulation (EC) No 1881/2006, the maximum level for OTA in cereal flour is 5 ng/g. A stock solution and a diluted solution (125 ng/ml) were produced from solid toxin and kept at −20°C. Then, 1.0 ml diluted solution which had 125 ng mycotoxin was sprayed to 25.0 g of rice sample at ambient temperature. After evaporation of the solvent, the sample was stirred to provide a good distribution of OTA in the rice (Heidari, Milani, & Nazari, 2014).

### 2.4 Washing process

After the rice sample infecting, washing process was performed after 24 hr. The rice samples were washed one, two, and three times in washing stage. Subsequently, the residue of OTA was measured in rice samples. The washing water volume was 100 ml, and washing time was 2 min at each stage.

### 2.5 Boiling process

After determining optimal washing conditions, the rice samples were boiled in a steel pot. Variable factors were boiling time (6–12 min), salt content (0%–7% w/w), and water‐to‐rice ratio (3:1 to 5:1). After cooking, the rice was rinsed by home strainer, and then, remaining OTA in the rice samples was extracted.

### 2.6 Extraction of OTA

Ground rice samples (25.0 g) were mixed with 100 ml methanol for 30 min. The extract was filtered through filter paper (Whatman No. 4), and 10 ml filtered solution was diluted with 40 ml phosphate‐buffered saline (PBS). A total of 50 ml of diluted extract was passed through the immunoaffinity column (IAC) at a flow rate of about 1 ml min^−1^. OTA was eluted from the column by passing 1.5 ml of HPLC‐grade methanol/acetic acid (98: 2, v/v). Then, 20 μl of eluate was injected into the HPLC (Heshmati & Mozaffari Nejad, 2015).

### 2.7 Statistical analysis

To evaluate changes in the levels of OTA, the data were statistically analyzed by one‐way analysis of variance (*p* < 0.05) and Duncan's test using SPSS software program, version 16 (SPSS Statistical Software, Inc., Chicago, IL).

Response surface methodology with a face‐centered central composite design (FCCD) with five central points was used to explore the effect of independent variables (salt content [0%–7% w/w], boiling time [6–12 min], and the ratio of water to rice [3:1 to 5:1 v/w]) on residue of OTA. Factor levels are presented in Table 1.

**Table 1:** Levels of independent variables in central composite design

Factor level	Boiling time (min)	Salt (%)	Water: rice ratio
−1	6	0	3
0	9	3.5	4
+1	12	7	5

Actual and coded levels of selected variables along with response variables are shown in Table 2. The software Statistica, version 7.0, was used to generate the experimental matrix and statistical calculation.

**Table 2:** Face‐centered central composite design for the independent variables (coded levels)

Run#	Area of OTA	Coded level of independent variables		
		Boiling time (min)	Salt content (%)	Water: rice
1	9,271	−1	−1	−1
2	12,380	−1	−1	+1
3	10,956	−1	+1	−1
4	6,450	−1	+1	+1
5	5,385	+1	−1	−1
6	12,468	+1	−1	+1
7	8,638	+1	+1	−1
8	4,990	+1	+1	+1
9	16,974	−1	0	0
10	15,770	+1	0	0
11	11,856	0	−1	0
12	10,302	0	+1	0
13	12,864	0	0	−1
14	9,773	0	0	+1
15	12,193	0	0	0
16	11,367	0	0	0
17	11,379	0	0	0
18	11,148	0	0	0
19	11,022	0	0	0

A second‐order polynomial equation (Equation (1)) was used by the software to show the relationship between response (OTA residue) and independent variables denoted by x_1_, x_2_, and x_3_ that were considered as salt content, boiling time, and water‐to‐rice ratio, respectively. β_0_, β_1_, β_2_, β_3_, β_11_, β_22_, β_33_, β_12_, β_13_, and β_23_ are the regression coefficients.

(1) $$Y=\beta_{0}+\sum_{j=1}^{k}\beta_{j}x_{j}+\sum_{j=1}^{k}\beta_{\mathrm{jj}}x_{j}^{2}+\sum_{i<}\sum_{j=2}^{k}\beta_{\mathrm{ij}}x_{i}x_{j}+\varepsilon$$

Statistical significance of the terms in the regression equations was investigated. The significant terms in the model for a response were found by analysis of variance (ANOVA) and determining the probability level in 95% significance.

## 3 RESULTS AND DISCUSSION

### 3.1 Method validation

Limit of detection (LOD), limit of quantification (LOQ), linear range, repeatability of retention times, and peak area for OTA were determined and are shown in Table 3. LOD and LOQ were calculated on the basis of 3S_b_/m and 10S_b_/m, respectively, where “S_b_” is the standard deviation of blank, and “m” is the slope of calibration curve. The calibration curves were obtained by triplicate injection of standard solutions.

**Table 3:** Validation data for the determination of OTA by HPLC

Parameters	
LOD (μg/kg)	0.13
LOQ (μg/kg)	0.39
Linear range (μg/kg)	2–250
Square of correlation coefficient (*r* ^2^)	0.88
Repeatability peak area, RSD (%, *n* = 5)	2.3
Repeatability retention time, RSD (%, *n* = 5)	0.9
%Recovery ± *SD*	97.0 ± 4.1

Note.

RSD, relative standard deviation; *SD*, standard deviation.

Method precision was determined by five replicate measurement of OTA in rice sample. To evaluate the accuracy of the method, rice sample was spiked with 5.0 μg/kg of OTA and the percentage recovery of the OTA was measured. Recovery percentage of OTA in rice was 97% and is shown in Table 3.

### 3.2 Effect of washing process on OTA level

The effects of washing process on OTA levels are presented in Table 4. It can be seen that OTA levels were reduced after the first, second, and third time of washing steps, but no significant difference in OTA level was observed between the second and third time of washing, and 42.68% of OTA was removed in entire washing process.

**Table 4:** Ochratoxin A (OTA) levels and reductions (%) during washing process

Sample	OTA content (ng/g)	Reduction (%)
Contaminated rice	4.85 ± 0.05^a^	
One‐time washed sample	3.61 ± 0.08^b^	25.56
Two‐time washed sample	2.96 ± 0.09^c^	38.96
Three‐time washed sample	2.78 ± 0.08^c^	42.68

Note.

The same letters in each column are not significantly different at *p* < 0.05.

According to results obtained on the effect of washing process on OTA content, the most current washing habit in Iranian cooking method (three‐time washing) was selected for investigating the effect of cooking process for the next stage.

Effect of washing and cooking process on aflatoxin B_1_ in two wheat products was investigated by Hwang and Lee (2006). They concluded that reduction in AFB_1_ was directly proportional to washing time. The concentration of AFB_1_ was reduced more by heating than washing treatment. In relation to cooking method, the reduction in hot water was more than steam cooking. This study confirms that moisture is an important factor to reduce AFB_1_ by heating.

Iha et al. (2009) studied the effect of different processes on the OTA content in dried beans. The results showed that washing had little effect on the OTA levels retained in whole beans. After cooking in water, about 45% reduction was seen. These findings are in agreement with our results, which confirms more important role of cooking process in comparison with washing on OTA residue.

Van der Westhuizen et al. (2011) studied the impact of sorting and washing of maize on reduction in fumonisin contamination under laboratory‐controlled conditions. Hand washing of the sorted kernels for a period of 10 min at ambient temperature resulted in 13% reduction. More reduction in OTA in current study, 42.68% for 6 (3 time*2 min) min, could be related to the soft texture of rice in comparison with maize as well as the waxy epidermis of maize kernels.

Effects of washing and drying on deoxynivalenol and zearalenone levels in wheat were investigated by Yener and Koksel (2013). Using chlorinated water, sodium carbonate and sodium hydroxide solutions for 1 min decreased deoxynivalenol levels in the range of 37.3%–91.2% and zearalenone levels in the range of 31.6%–83.6%. The results of this study indicated that pressure washing and microwave and infrared drying are promising methods for decontamination of wheat grains, even at high mycotoxin concentrations.

### 3.3 Effect of boiling process on OTA

The effects of boiling time, salt content, and water‐to‐rice ratio on the OTA level are given in Table 5.

**Table 5:** Linear, quadratic, interaction terms, and coefficients for the prediction models of OTA residue

Factor	Coefficient	*p*	*F*	Effect	*SD*
A	−878.03	0.003703	36.9399	−1756.06	144.4649
A^2^	231201	0.001117	69.9841	‐	276.3696
B	−1002.40	0.002266	48.1457	−2004.80	144.4649
B^2^	−2981.31	0.000419	116.3676	‐	276.3696
C^2^	−2741.81	0.000580	98.4221	‐	276.3696
AC	604.00	0.020129	13.9842	1208.00	161.5167
BC	−2293.25	0.000143	201.5896	−4586.50	161.5167

A, boiling time; B, salt content; C, water‐to‐rice ratio; *SD*, standard deviation.

The results showed that OTA content was mainly influenced by boiling time and salt content. Statistical analysis of data showed that boiling time and salt content had significant quadratic and linear effects on the response, but the ratio of water to rice had only quadratic effect. The mutual interactions between boiling time and water‐to‐rice ratio, and between salt content and water‐to‐rice ratio were also found to be significant.

The *R* ^2^ value was 0.829 for OTA content. A high *R* ^2^ indicates that the variation was accounted and the data fitted satisfactorily to the second‐order polynomial model. However, a large value of *R* ^2^ does not always imply that the regression model is a good one. Adding a variable to the model will always increase *R* ^2^, regardless of whether the additional variable is statistically significant or not. Thus, it is preferred to use an adj‐*R* ^2^ to evaluate the model adequacy, which should be over 0.5. Table 6 shows that *R* ^2^ and adj‐*R* ^2^ values for the models did not differ dramatically indicating that no significant term has been included in the model.

**Table 6:** ANOVA table of the regression model for ochratoxin A (OTA) residue

Source	*df*	Sum of squares	*F*
Model	7	138,205,953	7.65 > 3.01
Residual	11	28,385,622	
Lack of fit	7	18,545,118	6.08 < 6.09
Pure error	4	1,740,504	
Total	18	166,591,575	
*R* ^2^	0.82961		
Adj‐*R* ^2^	0.65922		

**Figure 1:**
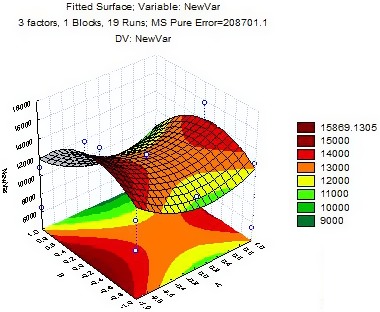
Effect of boiling time and water ratio on OTA residue in rice

As shown in Figure 1, the OTA level was firstly decreased by increasing boiling time within the studied variable ranges (from 0 to 9.6 min) and then increased (from 9.6 to 12). The decrease in OTA level may be due to concentration gradient of OTA content between rice and water which causes OTA molecules move from rice to water, which results in a decrease in OTA level during 9.6 min boiling in rice. After 9.6 min, the amount of OTA was started to increase, which is probably due to the increase in the absorbed water containing OTA by rice due to starch gelatinizing.

**Figure 2:**
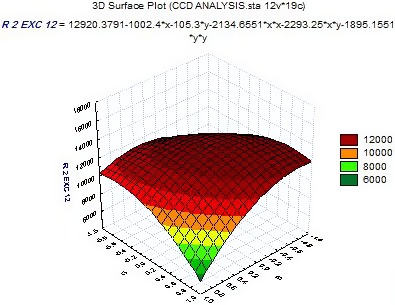
Effect of salt content and water ratio on OTA residue in rice

The effect of salt content and the ratio of water to rice on OTA is given in Figure 2. The results show that OTA level decreases with increasing salt content. The greatest reduction in toxin residue was observed in the amount of 7% w/w of salt. OTA is a weak organic acid, so increasing the salt concentration caused the interaction between salt ions and water increased, which led to the presence of greater toxins in the water and reducing toxin level in rice after rinsing.

Park et al. (2005) confirmed that cooking lowers OTA residues. Although washing polished rice with water had little effect on OTA levels, pressure steaming seemed to be the critical cooking step to reduce OTA residues in polished rice.

The effect of cooking process in carioca beans was investigated by Iha et al. (2009). Dried beans were washed with water for 2, 60, or 120 min, soaked in water for 60, 120 min, or 10 hr, and cooked for 60 or 120 min. The combination of the three treatments eliminated about 50% of the toxin from whole beans, and OTA was removed by 45% from beans by cooking (Iha et al., 2009).

Oliveira et al. (2013) studied the effects of roasting and particle size on the residue concentration of OTA in roasted and ground coffee. The beans were roasted to three levels (light, medium, and dark) and ground into three types (fine, medium, and coarse) after an incubation period. The combination of dark roast and coarse particle size had the lowest concentration of OTA, 3.06 mg/kg with a 97.17% reduction (Oliveira et al., 2013).

### 3.4 Optimization

In this study, optimum condition for the least amount of OTA in the rice was examined among the levels of salt amount, water:rice and boiling time in the range of 0%–7%, 3:1 to 5:1, and 6–12 min, respectively. In the other words, the optimum condition was obtained at 3.5% salt, 5:1 ratio of water to rice, and 9.6 min boiling time. These conditions caused the lowest level of OTA in rice, which it was 0.72 (ng/g). Optimal condition was determined by the numerical solution of regression model in the specific range of each independent variable via a grid search program written in Excel. Results showed that the amount of OTA remaining in rice was 1.05 (ng/g). Therefore, the optimum conditions of washing and cooking reduced 76% of OTA in rice.

## 4 CONCLUSIONS

The effect of washing and cooking processes indicated significant reduction in OTA levels. The results showed that washing stage was effective on OTA while there was no significant difference between second and third time washing. In boiling stage, effect of boiling time, salt content, and water‐to‐rice ratio was studied on the amount of remained mycotoxin in rice. According to the results, optimum condition for a boiling time, the amount of salt, and water‐to‐rice ratio were determined as 9.6 min, 3.5% salt, and 4:1 water‐to‐rice ratio, respectively. Therefore, the preparation process of rice including washing, boiling, and rinsing reduced OTA in rice.

## CONFLICT OF INTEREST

The authors declare that they do not have any conflict of interest.

## ETHICAL REVIEW

This study was approved by Sari Agricultural Sciences and Natural Resources University. This study does not involve any human or animal testing.

## INFORMED CONSENT

Written informed consent was obtained from all study participants.
